# Immunological analysis and differential genes screening of venous thromboembolism

**DOI:** 10.1186/s41065-020-00166-6

**Published:** 2021-01-02

**Authors:** Li-Na Gao, Qiang Li, Jian-Qin Xie, Wan-Xia Yang, Chong-Ge You

**Affiliations:** 1 Laboratory Medicine Center, Lanzhou University Second Hospital, Lanzhou, 730030 China; 2 Department of Gastroenterology, The First Hospital of Lanzhou University, Lanzhou, 730030 China; 3 Department of Anesthesiology, Lanzhou University Second Hospital, Lanzhou, 730030 China

**Keywords:** Venous thromboembolism, Immune cell, Differentially expressed genes, Ribosome, Immune differential gene, Bioinformatics analysis

## Abstract

**Purpose:**

To explore the pathogenesis of venous thromboembolism (VTE) and provide bioinformatics basis for the prevention and treatment of VTE.

**Methods:**

The R software was used to obtain the gene expression profile data of GSE19151, combining with the CIBERSORT database, obtain immune cells and differentially expressed genes (DEGs) of blood samples of VTE patients and normal control, and analyze DEGs for GO analysis and KEGG pathway enrichment analysis. Then, the protein-protein interaction (PPI) network was constructed by using the STRING database, the key genes (hub genes) and immune differential genes were screened by Cytoscape software, and the transcription factors (TFs) regulating hub genes and immune differential genes were analyzed by the NetworkAnalyst database.

**Results:**

Compared with the normal group, monocytes and resting mast cells were significantly expressed in the VTE group, while regulatory T cells were significantly lower. Ribosomes were closely related to the occurrence of VTE. 10 hub genes and immune differential genes were highly expressed in VTE. MYC, SOX2, XRN2, E2F1, SPI1, CREM and CREB1 can regulate the expressions of hub genes and immune differential genes.

**Conclusions:**

Ribosomal protein family genes are most relevant to the occurrence and development of VTE, and the immune differential genes may be the key molecules of VTE, which provides new ideas for further explore the pathogenesis of VTE.

## Background

Venous thromboembolism (VTE), including deep vein thrombosis and pulmonary embolism, is a highly prevalent and potentially fatal disease that causes 3 million deaths worldwide every year [1]. VTE is a common cancer complication [2]. The risk of venous thrombosis in cancer patients is as high as 7% due to their hypercoagulability or surgical intervention [3], which has become the second leading cause of death in cancer patients [4]. Compared with healthy people, the incidence of VTE in malignant tumor patients is 4–7 times higher, with a high incidence of morbidity and mortality [5]. From 2004 to 2016, the incidence of VTE in China was on the rise, increased from 34.8% in 2005 to 60.9% in 2014 [6]. In etiology, the most common cause of VTE is active malignant tumors. Therefore, it is important to study the pathogenesis of VTE to reduce the risk of postoperative death in cancer patients. Previous studies have generally believed that the occurrence of VTE is mainly related to the activation of coagulation function, slow blood flow, vascular wall injury, and its treatment uses anticoagulants, and thrombolytic drugs to prevent thrombus expansion and reduce thrombus. Nevertheless, in many VTEs, tissue factors reflecting vascular endothelial cell injury, P selectin activated by platelets did not rise significantly, which is difficult to explain with the traditional theory. It is reported that VTE is a complex disease influenced by environment [7], genetics [8] and epigenetics [9], which play important roles in the occurrence of VTE. A number of genes have been found to be susceptible to VTE occurrence [10, 11]. Recently, some scholars have shown that immune gene mutations may provide new insights into the pathogenesis of VTE [12]. However, the specific pathogenesis of VTE is still unclear.

Ribosomal protein is an important component of ribosomes. Abnormal expression of ribosomal protein gene will seriously affect cell growth, proliferation and differentiation. It has been found that the ribosomal proteins synthesis is closely related to the growth and proliferation of tumor cells during tumorigenesis [13, 14]. Recently, studies have found that ribosomes and immune-related genes are associated with systemic vasculitis [15]. However, studies on ribosomal proteins, immune-related genes and VTE are rarely reported. This study enriched the function of genes with different expression in VTE through bioinformatics, and obtained important pathways and molecules involved in the development process of VTE, which provided bioinformatics basis for the research on the mechanism of VTE occurrence and development, and also provided new potential targets for the research on the treatment of VTE.

## Materials and methods

### Microarray data

This study downloaded the microarray chip GSE19151 from the GEO database (https://www.ncbi.nlm.nih.gov/geo/). The chip contains a total of 133 blood samples were obtained from 70 VTE patients and 63 healthy controls. The chip platform is GPL571 [HG-U133A_2] Affymetrix Human Genome U133A 2.0 Array.

### Obtaining immune cells

The R language was used to conduct background correction, add missing value and remove duplicate of the original data. The probe name of the chip GSE19151 matrix data was converted into the gene name through the platform file GPL571. The CIBERSORT database (http://cibersort.stanford.edu) provides a calculation method for quantifying cell composition from gene expression profiles of a large number of tissues and can estimate the immune composition of the sample. CIBERSORT was used to convert the gene expression profile data into the corresponding immune cell proportion data of the samples, run with 100 permutations and a threshold of *P* < 0.05. Then the Perl was used to filter the data and delete the samples with *P* > 0.05. The R software pheatmap and vioplot package were used to draw the heatmap and vioplot of the immune cell distribution in the samples.

### Obtaining differentially expressed genes (DEGs)

The Limma package was use to screen DEGs, the screening criterion was set as | log_2_FC |> 1 and adjusted *P* < 0.05. The R software is used to draw heatmap and volcano plot of DEGs.

### Enrichment analysis of DEGs

Gene Ontology (GO) includes biological process (BP), cellular component (CC), and molecular function (MF). To understand the functions of DEGs, we used the R software clusterProfiler package to conduct GO enrichment analysis and Kyoto Encyclopedia of Genes and Genomes (KEGG) pathway analysis on DEGs, and drew bubble plot for GO analysis and network plot for KEGG pathway. A adjusted *P* < 0.05 was considered as statistically significant.

### PPI network construction and hub genes screening

STRING (Search Tool for the Retrieval of Interacting Genes, https://string-db.org/) database [16] can construct protein-protein interaction network to evaluate functional genomics data. We used STRING online database to construct PPI network of DEGs, the obtained source files were imported into Cytoscape software for visual analysis and hub gene screening. Meanwhile, MCC algorithm was used to select the top 10 hub genes.

### Immune differential genes screening

To further understand the immune genes involved in VTE, the immune-related genes were obtained through the IMMPORT (https://www.immport.org/) database [17], which consists of four components-Private Data, Shared Data, Data Analysis, and Resources for data archiving, dissemination, analyses, and reuse. The VTE immune differential genes were screened in VTE DEGs. The screening criterion was set as | log_2_FC | > 1 and adjusted *P* < 0.05.

### TF-Hub- and immune genes network establishment

NetworkAnalyst (http://www.networkanalyst.ca) database [18] is a comprehensive visualization analysis platform for gene expression profiling and meta-analysis designed to meet the demands of PPI networks to interpret gene expression data. It can conduct gene expression profile analysis of 17 different species. In addition to the general PPI network, it can also create cell type or tissue specific PPI networks, gene regulation networks, gene co-expression networks, and pharmacogenomics research networks. To further understand the TFs that regulate the hub gene and immune differential genes, we obtained the TFs of regulating hub gene through the NetworkAnalyst database based on the data provided on ChEA.

## Results

### Immune cells in VTE and normal samples

**Fig. 1:**
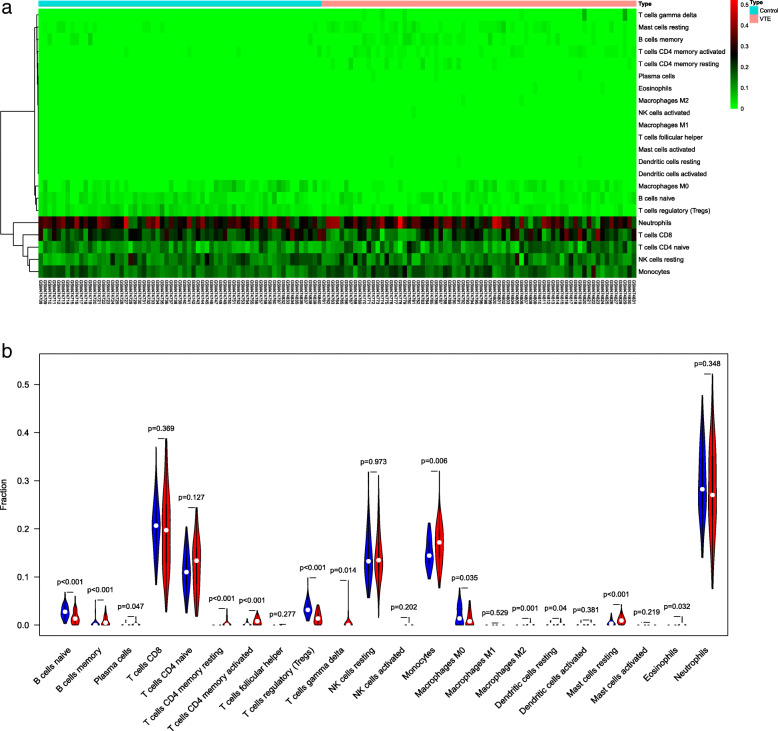
Immune cells in VTE and normal control. **a** Heatmap analysis of the 22 immune cells. The horizontal axis shows the samples are divided into normal control and VTE group. **b** Vioplot of immune cells. The blue and red violin represent the normal control and the VTE group, respectively

Converting the gene expression profile data into the corresponding immune cell proportion data of the samples, we obtained the difference in the distribution of the immune cells in the VTE and normal group. The results showed that compared with the normal group, monocytes and resting mast cells were higher in number in the VTE group, while regulatory T cells were significantly lower (*P* < 0.05), as shown in Fig. 1a and b.

### Screening of differentially expressed genes

**Fig. 2:**
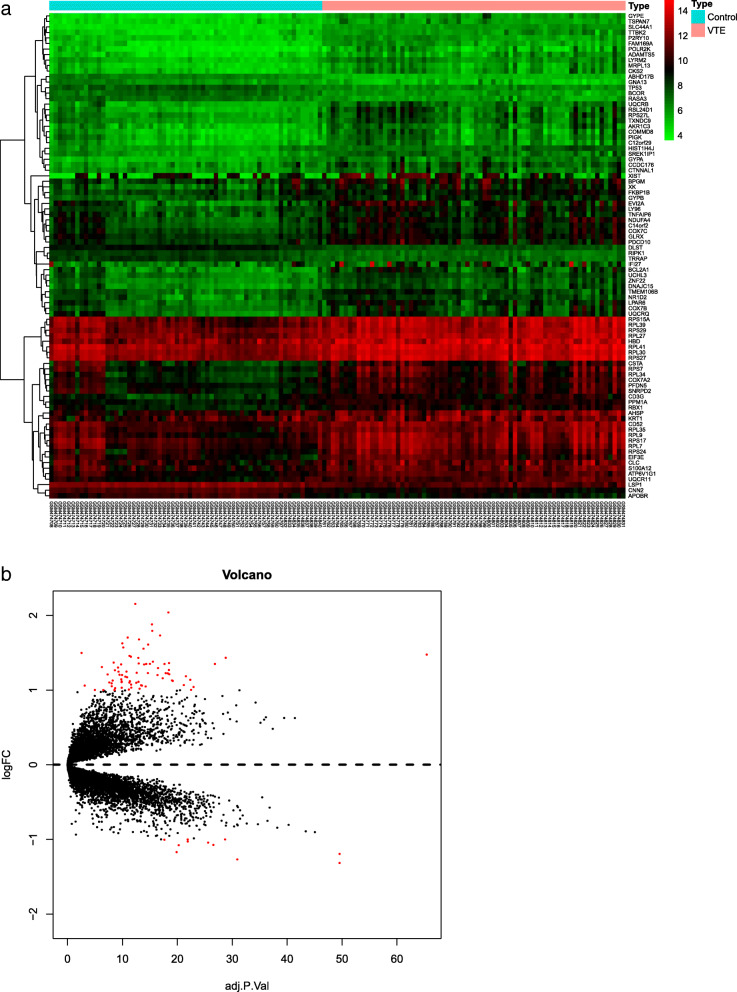
DEGs in the VTE and normal group. **a** Heatmap analysis of differential genes. The red and green represent the significantly upregulated and downregulated DEGs. **b** Volcano plot of the differentially expressed genes. These genes consist of 77 upregulated genes and 11 downregulated genes. The screening criterion was | log_2_FC |> 1 and adjusted *P* < 0.05

To further investigate the intrinsic differences in immunity between VTE and normal samples, we performed a bayes test on GSE19151 gene chip data, and obtained a total of 88 DEGs (VTE group/normal control group), of which 77 were upregulated and 11 were downregulated. The expression of the differential genes in the two groups is shown in Fig. 2a and b.

### Function and pathway enrichment analyses of DEGs

**Fig. 3:**
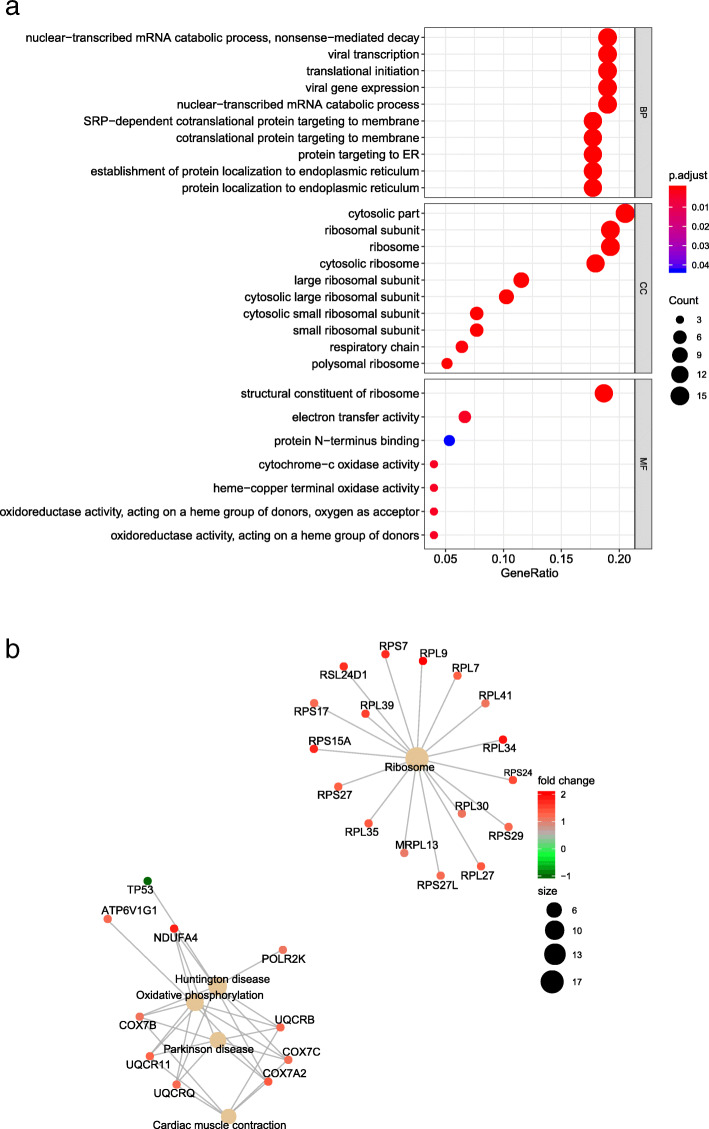
Enrichment analysis of DEGs. **a** GO function of DEGs. **b** The pathways enriched for DEGs in VTE. The size of the circle indicates the number of genes. The screening criterion was set as adjusted *P* < 0.05

The R language was used to perform functional enrichment analysis on 88 DEGs, and the results were shown in Fig. 3a. DEGs are mainly involved in the composition of ribosomes and respiratory chains in the cytoplasm. The biological process involves nuclear transcription mRNA metabolism, translation initiation, and endoplasmic reticulum protein localization. The molecular function mainly focus on ribosome composition, cytochrome C oxidase activity, and oxidoreductase activity. KEGG pathway enrichment analysis showed that the DEGs are mainly involved ribosomes, oxidative phosphorylation, Huntington disease, Parkinson disease, myocardial contraction and other processes (Fig. 3b).

### PPI network analysis and hub gene screening of DEGs

**Fig. 4:**
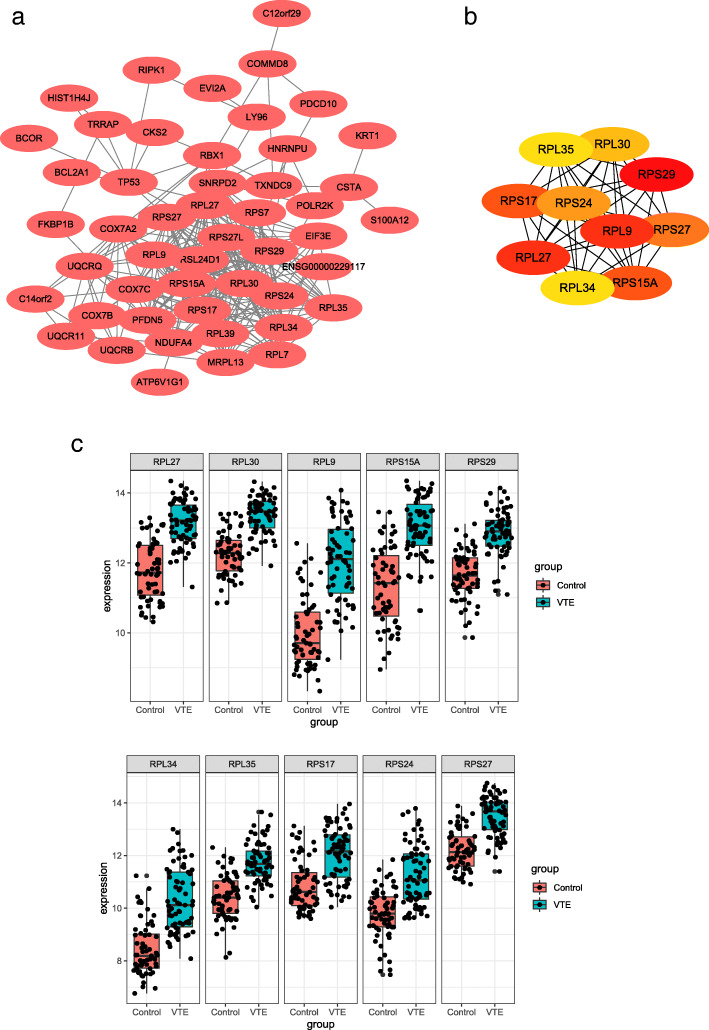
PPI network construction among DEGs and top 10 hub genes. **a** PPI network construction among DEGs. **b** The top 10 hub genes. **c** The expression of hub genes in VTE group and normal control group

The 88 DEGs were input into the STRING database, and the obtained data were imported into Cytoscape to construct PPI network(Fig. 4a) and to find the top 10 hub genes, which were RPS29, RPL9, RPL27, RPS15A, RPS17, RPS27, RPS24, RPL30, RPL34 and RPL35 (Fig. 4b). The expression differences of these 10 hub genes in the VTE group and the normal control group are shown in Fig. 4c.

### Immune differential genes screening

**Fig. 5:**
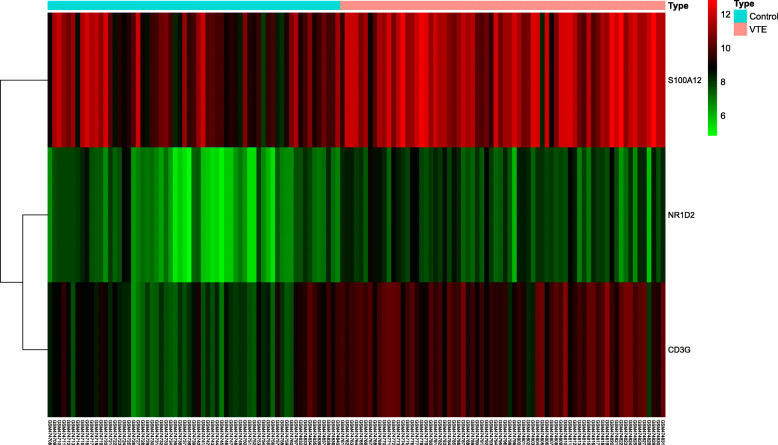
The expression of immune differential genes in VTE group and normal control group

To further clarify the relationship between VTE and immune genes, we combined the selected DEGs and the immune genes of the IMMPORT database to obtain a total of 3 immune differential genes S100A12, NR1D2 and CD3G. Compared with the control group, S100A12, NR1D2 and CD3G were significantly overexpressed in the VTE group. The results are shown in Fig. 5.

### Analysis of TFs regulatory network of DEGs

**Fig. 6:**
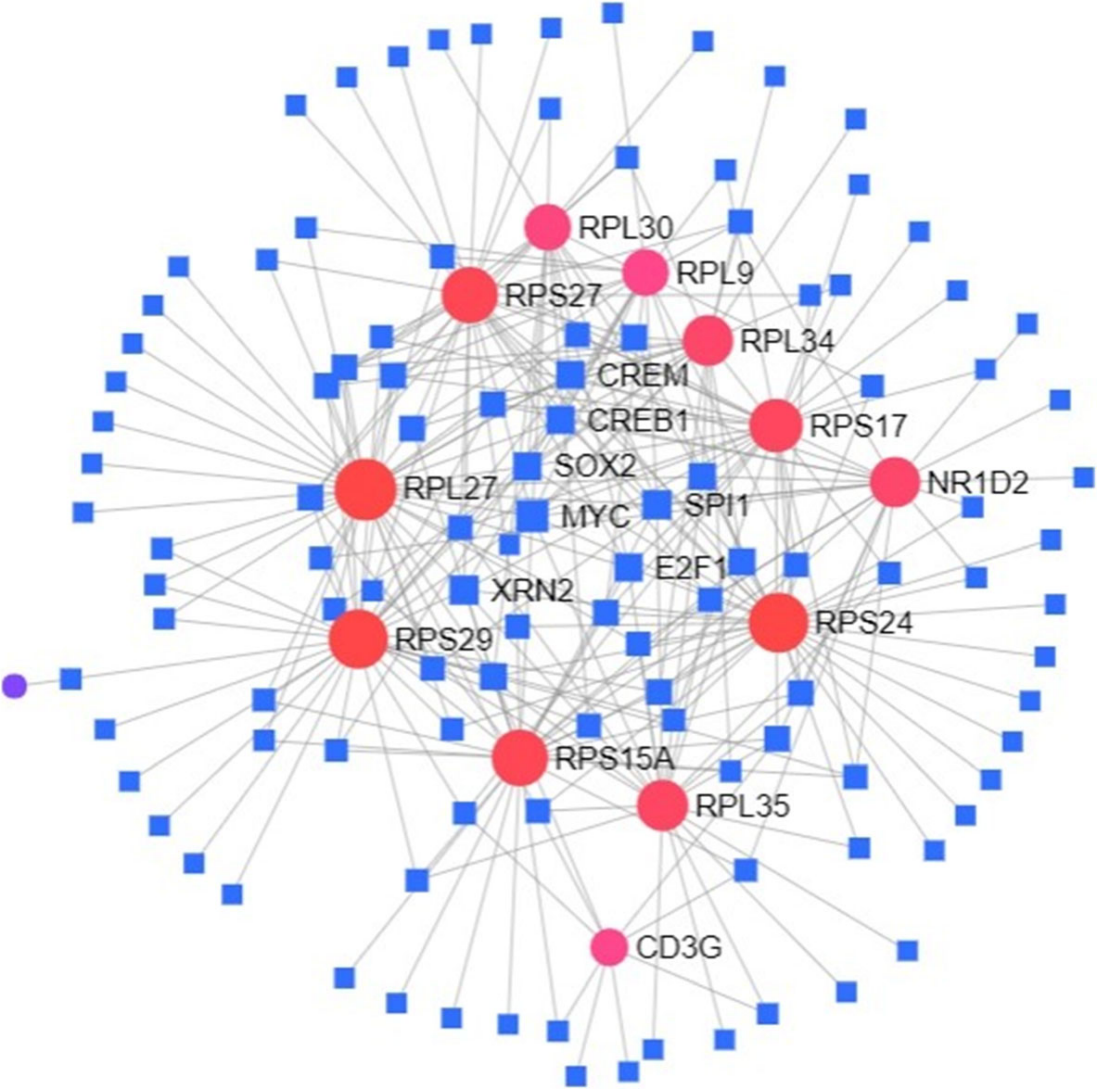
The network of TFs and differential genes

To further study the molecules that regulate 10 hub genes and three immune differential genes S100A12, NR1D2 and CD3G, this study screened the potential regulatory relationship between TFs and DEGs based on the data provided on ChEA, and predicted TFs that can regulate the expression of hub genes and immune differential genes. It was found that MYC, SOX2, XRN2, E2F1, SPI1, CREM and CREB1 can regulate the expression of these 13 genes (Fig. 6).

## Discussion

VTE is a systemic disease. A study of more than 1 million cancer patients showed that the incidence of VTE increased by 28% between 1995 and 2003 [19]. In addition, among VTE patients, cancer patients can account for up to 20%, so it is crucial to study the mechanism of VTE. Mauracher LM et al. [20] studied the mechanism of cancer-related VTE and found that biomarkers formed by Neutrophil extracellular traps (NETs) are related to the occurrence of VTE in cancer patients, and can become a new pathogenic mechanism reflecting cancer-related VTE. At present, although a lot of work has been devoted to the study of VTE molecular mechanism, the related research is still in the exploratory stage. Therefore, the research on VTE related molecular mechanisms can not only re-understand the pathogenesis of VTE, but also provide new ideas for clinical treatment. Omics and bioinformatic algorithms, mostly network-oriented, are one of the most effective ways to capture new pathways of cardiovascular disease [21, 22]. Based on this, the molecular level analysis of VTE was carried out by bioinformatics to provide new targets for the prevention and treatment of VTE.

The immune cell analysis of this study showed that monocytes and resting mast cells were significantly overexpressed in VTE, while regulatory T cells were significantly lower expressed. A study suggested that monocyte-related inflammatory mechanisms may be involved in the pathophysiological processes of VTE [23]. Monocytes, platelet aggregation, and C-reactive protein are associated with VTE [24], which supports the conclusion that VTE is associated with inflammation. GO enrichment analysis found that DEGs are mainly involved in the composition of ribosomes in the cytoplasm. The occurrence of VTE is related to the ribosomes, cytochrome C oxidase activity, and the oxidoreductase activity on heme. It has been reported that the heme released by the large amount of erythrolysis in early thrombus is the main source of vascular endothelial cell oxidation, which can further lead to endothelial cell dysfunction and thrombosis [25, 26]. Since heme is an inflammatory substance, the inflammatory process is increasingly recognized as an important mechanism for regulating thrombosis [27]. In addition, heme can also promote the transformation of LDL into cytotoxic oxidation products. Elevated oxidized LDL is a known risk factor for cardiovascular and cerebrovascular diseases, and it is also a common independent risk factor for VTE [28]. As can be seen, the oxidoreductase acting on heme plays an important role in VTE.

KEGG pathway enrichment analysis showed that DEGs are mainly involved in ribosomes, oxidative phosphorylation, Huntington’s disease, Parkinson’s disease, myocardial contraction and other processes. In addition, the 10 hub genes RPS29, RPL9, RPL27, RPS15A, RPS17, RPS27, RPS24, RPL30, RPL34 and RPL35 screened from DEGs all belong to the ribosomal protein family, and they are all highly expressed in VTE. Studies have shown that cardiovascular and cerebrovascular diseases are important risk factors for VTE [29], and ribosomes can be used as targets for cardiovascular diseases [30]. Therefore, it can be speculated that there is a certain connection between them. In addition, scholars have found that ribosomes play an important role in the translation of platelet proteins [31], and platelets can promote the formation of NETs, which in turn promotes the formation of deep vein thrombosis [32]. This conclusion supports our speculation. However, there is little direct evidence of ribosomal protein family genes associated with thrombosis.

Inflammation is one of the initial responses of the immune system to stimulus. Studies have found that immune dysfunction may be associated with the occurrence of VTE [33]. Recently, scholars can use immunology to explain some “unprovoked” VTE cases [34]. In order to further clarify the relationship between VTE and the immune system, we selected three immune differential genes S100A12, NR1D2 and CD3G from DEGs, which were significantly overexpressed in VTE. Calcium binding protein S100A12 is an immune molecule present on the mucosal surface, which is mainly secreted by neutrophils [35]. In addition, an increase in the number of neutrophils can increase the risk of VTE events [36]. A cross-sectional study of 550 hemodialysis patients showed that S100A12 protein levels were closely related to the prevalence of cardiovascular disease [37]. Therefore, S100A12 can be considered as a biomarker for inflammatory diseases such as VTE. Studies have found that genetic defects of CD3G can lead to impaired expression of CD3 and TCR on the cell surface, so CD3G mutations are related to immune function defects [38]. CD3G gene polymorphism may affect the occurrence of liver cancer [39]. At present, most of the research on CD3G focuses on tumors, and there are few reports on vascular diseases and thrombosis. NR1D2, a nuclear hormone receptor gene, is associated with circadian rhythm, and circadian dysregulation widely affects Ras signaling pathways, T cell receptor signaling pathways, and then triggers various diseases such as tumors [40]. NR1D2 is also an important regulator of vascular inflammation and is related to the occurrence of cardiovascular events [41]. As a common complication of tumor, VTE may be closely related to immune genes CD3G and NR1D2.

Compared with previous studies, this article focuses on the genetics and immunology of VTE, and finds that ribosomal protein family genes and immune-related genes are closely related to VTE, which provides new ideas for further exploring the pathogenesis of VTE. Of course, this study also has some limitations. First, the chip data in this study is a single-center study with limitations in representativeness. Second, the molecular mechanism and therapeutic targets of VTE still need to be verified by a series of experiments.

## Conclusions

In summary, this study use bioinformatics analysis to find that the occurrence of VTE is related to the abnormal expression of monocytes and mast cells resting. Ribosomes play a vital role in the occurrence of VTE. The ribosomal protein family genes may be potential therapeutic targets for the treatment of VTE. In addition, immune genes S100A12, NR1D2 and CD3G may also be effective targets for the prevention and treatment of VTE, providing new ideas for the mechanism and treatment of VTE. It can be seen that the occurrence of VTE is related to inflammation, abnormal gene expression and immune abnormalities.

## Data Availability

The data used to support the findings of this study are included within the article.

## Abbreviations

VTE: Venous thromboembolism
DEG: Differentially expressed gene
GO: Gene ontology
KEGG: Kyoto Encyclopedia of Genes and Genomes
BP: Biological process
CC: Cellular component
MF: Molecular function
PPI: Protein–protein interaction
STRING: The Search Tool for the Retrieval of Interacting Genes
TFs: Transcription factors

## References

[1] Fernandes C, Jardim CVP, Alves JL (2018). Reperfusion in acute pulmonary thromboembolism. J Bras Pneumol.

[2] Kraaijpoel N, Carrier M (2019). How I treat cancer-associated venous thromboembolism. Blood.

[3] Stricker H (2014). Venous thromboembolism and cancer: pathophysiology and incidence. Vasa.

[4] Farge D, Bounameaux H, Brenner B (2016). International clinical practice guidelines including guidance for direct oral anticoagulants in the treatment and prophylaxis of venous thromboembolism in patients with cancer. Lancet Oncol.

[5] Navi BB, Reiner AS, Kamel H (2017). Risk of arterial thromboembolism in patients with cancer. J Am Coll Cardiol.

[6] Huang D, Chan PH, She HL (2018). Secular trends and etiologies of venous thromboembolism in Chinese from 2004 to 2016. Thromb Res.

[7] Heit JA (2015). Epidemiology of venous thromboembolism. Nat Rev Cardiol.

[8] Klarin D, Busenkell E, Judy R (2019). Genome-wide association analysis of venous thromboembolism identifies new risk loci and genetic overlap with arterial vascular disease. Nat Genet.

[9] Benincasa G, Costa D, Infante T (2019). Interplay between genetics and epigenetics in modulating the risk of venous thromboembolism: A new challenge for personalized therapy. Thromb Res.

[10] Lindstrom S, Wang L, Smith EN (2019). Genomic and transcriptomic association studies identify 16 novel susceptibility loci for venous thromboembolism. Blood.

[11] Ruhle F, Witten A, Barysenka A (2017). Rare genetic variants in SMAP1, B3GAT2, and RIMS1 contribute to pediatric venous thromboembolism. Blood.

[12] Duan Q, Lv W, Yang M (2015). Characterization of immune cells and perforin mutations in familiar venous thromboembolism. Int J Clin Exp Med.

[13] Barna M, Pusic A, Zollo O (2008). Suppression of Myc oncogenic activity by ribosomal protein haploinsufficiency. Nature.

[14] Dong Z, Jiang H, Liang S (2019). Ribosomal protein L15 is involved in colon carcinogenesis. Int J Med Sci.

[15] Gan SJ, Ye B, Qian SX (2015). Immune- and ribosome-related genes were associated with systemic vasculitis. Scand J Immunol.

[16] Szklarczyk D, Morris JH, Cook H (2017). The STRING database in 2017: quality-controlled protein-protein association networks, made broadly accessible. Nucleic Acids Res.

[17] Bhattacharya S, Andorf S, Gomes L (2014). ImmPort: disseminating data to the public for the future of immunology. Immunol Res.

[18] Zhou G, Soufan O, Ewald J (2019). NetworkAnalyst 3.0: a visual analytics platform for comprehensive gene expression profiling and meta-analysis. Nucleic Acids Res.

[19] Khorana AA, Francis CW, Culakova E (2007). Frequency, risk factors, and trends for venous thromboembolism among hospitalized cancer patients. Cancer.

[20] Mauracher LM, Posch F, Martinod K (2018). Citrullinated histone H3, a biomarker of neutrophil extracellular trap formation, predicts the risk of venous thromboembolism in cancer patients. J Thromb Haemost.

[21] Silverman EK, Schmidt H, Anastasiadou E (2020). Molecular networks in network medicine: development and applications. Wiley Interdiscip Rev Syst Biol Med.

[22] Benincasa G, Marfella R, Della Mura N (2020). Strengths and opportunities of network medicine in cardiovascular diseases. Circ J.

[23] Wypasek E, Padjas A, Szymanska M (2019). Non-classical and intermediate monocytes in patients following venous thromboembolism: links with inflammation. Adv Clin Exp Med.

[24] Shih L, Kaplan D, Kraiss LW (2016). Platelet-monocyte aggregates and C-reactive protein are associated with VTE in older surgical patients. Sci Rep.

[25] Balla J, Vercellotti GM, Jeney V (2007). Heme, heme oxygenase, and ferritin: how the vascular endothelium survives (and dies) in an iron-rich environment. Antioxid Redox Signal.

[26] Woollard KJ, Sturgeon S, Chin-Dusting JP (2009). Erythrocyte hemolysis and hemoglobin oxidation promote ferric chloride-induced vascular injury. J Biol Chem.

[27] Saghazadeh A, Rezaei N (2016). Inflammation as a cause of venous thromboembolism. Crit Rev Oncol Hematol.

[28] Von Depka M, Nowak-Gottl U, Eisert R (2000). Increased lipoprotein (a) levels as an independent risk factor for venous thromboembolism. Blood.

[29] Yang CC, Kao CC (2007). Cardiovascular diseases and the risk of venous thromboembolism: a hospital-based case-control study. J Chin Med Assoc.

[30] Pellegrino S, Yusupova G (2016). Eukaryotic ribosome as a target for cardiovascular disease. Cell Chem Biol.

[31] Rowley JW, Weyrich AS (2017). Ribosomes in platelets protect the messenger. Blood.

[32] Von Bruhl ML, Stark K, Steinhart A (2012). Monocytes, neutrophils, and platelets cooperate to initiate and propagate venous thrombosis in mice in vivo. J Exp Med.

[33] Duan Q, Gong Z, Song H (2012). Symptomatic venous thromboembolism is a disease related to infection and immune dysfunction. Int J Med Sci.

[34] Vazquez-Garza E, Jerjes-Sanchez C, Navarrete A (2017). Venous thromboembolism: thrombosis, inflammation, and immunothrombosis for clinicians. J Thromb Thrombolysis.

[35] Pulsipher A, Davis BM, Smith KA (2018). Calgranulin C (S100A12) is differentially expressed in subtypes of chronic rhinosinusitis. Am J Rhinol Allergy.

[36] Rattazzi M, Villalta S, Galliazzo S (2013). Low CD34(+) cells, high neutrophils and the metabolic syndrome are associated with an increased risk of venous thromboembolism. Clin Sci (Lond).

[37] Shiotsu Y, Mori Y, Nishimura M (2011). Plasma S100A12 level is associated with cardiovascular disease in hemodialysis patients. Clin J Am Soc Nephrol.

[38] Rowe JH, Delmonte OM, Keles S (2018). Patients with CD3G mutations reveal a role for human CD3gamma in Treg diversity and suppressive function. Blood.

[39] Jiang L, Xu J, Ni J (2012). A functional insertion/deletion polymorphism in the proximal promoter of CD3G is associated with susceptibility for hepatocellular carcinoma in Chinese population. DNA Cell Biol.

[40] Wu Y, Tao B, Zhang T (2019). Pan-cancer analysis reveals disrupted circadian clock associates with T cell exhaustion. Front Immunol.

[41] Duez H, Staels B (2008). The nuclear receptors Rev-erbs and RORs integrate circadian rhythms and metabolism. Diab Vasc Dis Res.

